# Echocardiographic Indices in Patients with End-Stage Renal Disease and Their Association with Hemodialysis-to-Hemodiafiltration Transfer: A Prospective Observational Study

**DOI:** 10.3390/medicina60091537

**Published:** 2024-09-20

**Authors:** Josipa Domjanović Matetić, Darija Baković Kramarić, Tea Domjanović Škopinić, Ivo Jeličić, Dijana Borić Škaro, Joško Božić, Andrija Matetic

**Affiliations:** 1 Department of Nephrology, University Hospital of Split, 21000 Split, Croatia; josipa.domjanovic@gmail.com (J.D.M.); ivo.jelicic@gmail.com (I.J.); zanaboric@gmail.com (D.B.Š.); 2 Department of Cardiology, University Hospital of Split, 21000 Split, Croatia; darija.bakovic@mefst.hr (D.B.K.); tea.domjanovic@gmail.com (T.D.Š.); 3 Department of Pathophysiology, University of Split School of Medicine, 21000 Split, Croatia; josko.bozic@mefst.hr; 4 University of Split School of Medicine, 21000 Split, Croatia

**Keywords:** end-stage renal disease, hemodialysis, hemodiafiltration, contemporary echocardiography

## Abstract

*Background and Objectives*: The assessment of cardiac function in patients with end-stage renal disease (ESRD) is vital due to their high cardiovascular risk. However, contemporary echocardiographic indices and their association with hemodialysis-to-hemodiafiltration transfer are underreported in this population. *Materials and Methods*: This prospective cohort study enrolled 36 ESRD patients undergoing hemodialysis-to-hemodiafiltration transfer, with baseline and 3-month post-transfer comprehensive echocardiographic assessments. The key parameters included the global work index, global constructed work, global wasted work (GWW), global work efficiency (GWE), and global longitudinal strain (GLS), with secondary measures from conventional echocardiography. The baseline measures were compared to general population reference values and changes pre- to post-transfer were analyzed using the Mann–Whitney U test. *Results*: Patients exhibited significant deviations from reference ranges in GWW (179.0 vs. 53.0–122.2 mmHg%), GWE (90.0 vs. 53.0–122.2%), and GLS (−16.0 vs. −24.0–(−16.0)%). Post-transfer left ventricular myocardial work and longitudinal strain remained unchanged (*p* > 0.05), except for increased GWW (179.0, IQR 148.0–217.0 to 233.5, IQR 159.0-315.0 mmHg%, *p* = 0.037) and improved mid-inferior peak systolic longitudinal strain ((−17.0, IQR −19.0–(−11.0) to −18.7, IQR −20.0–(−18.0)%, *p* = 0.016). The enrolled patients also showed higher left atrial diameters, left ventricular volumes, and mass, with impaired systolic function in both ventricles compared to reference values. *Conclusions*: This study highlights baseline impairments in contemporary echocardiographic measures (GWW, GWE, GLS) in ESRD patients versus reference values, but found no association between hemodialysis-to-hemodiafiltration transfer and most myocardial work and strain parameters.

## 1. Introduction

End-stage renal disease (ESRD) is a major global health burden with substantial cardiovascular complications and increased cardiovascular mortality [1,2]. These patients exhibit several negative cardiovascular risk factors, such as accelerated atherosclerosis due to chronic inflammation [2], enhanced vascular and valvular calcifications resulting from impaired mineral metabolism [2,3], and cardiac dysfunction driven by increased loading conditions [4]. Additionally, negative cardiovascular effects may be associated with dialysis that carries proinflammatory response, intradialytic hypotension [5], cardiac load oscillations, and impaired myocardial perfusion [5,6].

As a result, there is a growing trend toward timely stratification of cardiovascular function in ESRD patients, but also toward transitioning patients to newer dialytic modalities like hemodiafiltration, which may offer cardiovascular benefits [1,7,8]. Hemodiafiltration allows a more effective removal of harmful proinflammatory and proatherogenic substances, such as β2-microglobulin, cytokines, advanced glycation end products, and fibroblast growth factors [9,10]. Although the removal of these middle-weight molecules could mitigate negative cardiovascular effects, their impact on cardiovascular tests such as echocardiography is still unclear.

Echocardiography is an essential diagnostic method in patients with ESRD due to its non-invasiveness and availability. Contemporary echocardiographic analyses such as myocardial work and strain imaging enable the detection of subtle cardiac changes and may potentially allow for earlier therapeutic intervention in this vulnerable population. Importantly, previous studies have reported an association of advanced echocardiographic measures such as the left ventricular strain with mortality in pre-dialysis [11], stable hemodialysis [12], and overall ESRD patients [13], with worse function being associated with impaired prognosis. Their predictive performance was shown to be superior to standard echocardiographic measures like the left ventricular ejection fraction [14], further emphasizing their clinical importance and the need for further evaluation amongst patients with ESRD. However, contemporary echocardiographic data are generally underreported in ESRD patients, and there is an unmet need for their better understanding, particularly in the context of established reference ranges and different dialysis modalities.

Therefore, this study aimed to describe the contemporary echocardiographic measures in patients with ESRD and determine their association with the hemodialysis-to-hemodiafiltration transfer, using the comprehensive echocardiographic analyses. Importantly, this study has an important hypothesis-generating purpose to support further research and stimulate the development of novel diagnostic and therapeutic modalities in these settings.

## 2. Materials and Methods

### 2.1. Ethical and Reporting Considerations

This study was approved by the Ethics Committee of the University Hospital of Split, Croatia (Date of approval: 28 May 2021; No. 2181-147/01/06/M.S.-21-02). All participants completed an informed consent form. All the proceedings and clinical research were performed with the ethical standards of the Declaration of Helsinki and its amendments. The Strengthening the Reporting of Observational Studies in Epidemiology (STROBE) checklist for observational studies is reported in Supplementary STROBE Statement.

### 2.2. Study Design and Participants

This longitudinal prospective observational study enrolled a total of 36 consecutive adult patients (≥18 years) with ESRD who were scheduled for a planned therapeutic hemodialysis-to-hemodiafiltration transfer at the Department of Nephrology and Hemodialysis at the University Hospital of Split over the period from September 2021 to January 2023. The main eligibility criteria for the hemodialysis-to-hemodiafiltration transfer were defined solely on the institutional protocol, including the: regular management with conventional hemodialysis for at least 6 months (3 times a week); stable efficiency parameters (minimal single-pool Kt/V for urea of 1.2); and availability of adequate vascular access. The exclusion criteria were as follows: known or established valvular heart disease; active malignant disease; and poor echocardiographic acoustic window. For the purpose of this research study, the authors did not consider any other eligibility/exclusion criteria. The flow diagram of the study is shown in Supplementary Figure S1.

### 2.3. Data Sources

Detailed patient data were collected from the medical records, while other measurements were performed according to applicable medical standards. All anthropometric measurements were determined according to the recommendations of the World Health Organization, including the measurement of body height, body weight, and body mass index (BMI) [15]. Importantly, all medication regimens were stably maintained during the study period.

Routine laboratory analysis was conducted according to the standard protocols of the Department of Nephrology and Hemodialysis at the University Hospital of Split. Venous blood was sampled by a trained medical technician/nurse from the dialytic system (up to 22 mL of blood) at different time points, including the last hemodialysis session (before and after the hemodialysis session) and 3 months after the hemodialysis-to-hemodiafiltration transfer (before and after the hemodiafiltration session).

### 2.4. Conventional Hemodialysis

Conventional hemodialysis was performed using the universal machine in our centre (Fresenius 5008; Fresenius SE & Co., Hochtaunuskreis, Germany) with poly-sulfone membranes and blood flow of 300 mL/min. The dialyzers used for conventional hemodialysis were Fx60 (Fresenius SE & Co., Hochtaunuskreis, Germany), Fx80 (Fresenius SE & Co., Hochtaunuskreis, Germany), Leoceed-16N (Asahi Kasei Corp., Tokyo, Japan), and Nipro 150 (Nipro Medical Europe, Mechelen, Belgium).

### 2.5. Hemodiafiltration

The on-line hemodiafiltration in this study was performed using the hemodiafiltration machine (Fresenius 5008) with poly-sulfone membranes containing >23 L of convective volume; blood flow of 300 to 350 mL/min; and dialysate flow of 500–600 mL/min. The dialyzers used for hemodiafiltration were Fx60 and Fx80.

Both hemodialysis and hemodiafiltration treatment modalities were performed using the ultrapure dialysates as defined by bacterial counts <0.1/mL colony-forming units (CFU) and <0.025 units of endotoxin per mL. 

### 2.6. Comprehensive Echocardiographic Assessment

The transthoracic echocardiographic examinations were performed by a single highly experienced cardiologist specialized in cardiac imaging, per standard echocardiographic protocols [16]. Echocardiographic imaging was conducted using the advanced ultrasound system (Vivid 9E; GE Healthcare, Chicago, IL, USA). Data were digitally stored and analyzed on the Echo PAC workstation (Echo PAC 202 PC; GE Healthcare, Chicago, IL, USA).

Echocardiography assessments were always performed on the non-dialysis days before the mid-week hemodialysis or hemodiafiltration. Measurements were collected at 2 time points: baseline measurement (before the hemodialysis-to-hemodiafiltration transfer), and control measurement (3 months after the hemodialysis-to-hemodiafiltration transfer). 

The echocardiographic analysis included standard 2-dimensional echocardiographic projections (parasternal long and short axis-view; apical 4, 5 and 3-chamber view) and additional modified views if necessary. Obtained images were used to measure standard 2-dimensional dimensions (left atrium, left ventricle, interventricular septum, posterior wall of the left ventricle, aortic root and ascending aorta) and to calculate 2-dimensional ejection fraction using Simpson’s biplane method. Left atrial volume and left ventricular mass were standardized according to body surface area (BSA). The 4-dimensional probe was used to obtain multi-slice images of the left ventricle and to derive 4-dimensional left ventricular volumes. Valves were analyzed morphologically and functionally using Doppler echocardiography. Using the pulse wave and tissue Doppler echocardiography, parameters of left ventricular diastolic function were evaluated—E and A wave velocity, E/A ratio, E wave deceleration time, and e and e’ velocity. These measurements were combined with left atrial indexed volume and tricuspid velocity to determine the degree of diastolic dysfunction. 

Using the apical projections, global longitudinal strain by speckle tracking echocardiography and myocardial work were determined to evaluate left ventricular function. Myocardial work is a pressure-dependent echocardiographic measure of left ventricular function that relies on the pressure–strain loop, thereby reducing its dependence on the afterload conditions [17]. It can be further divided into global work index (GWI), global constructive work (GCW), global wasted work (GWW), and global work efficiency (GWE). GWI represents total left ventricular work, GCW represents systolic shortening of cardiomyocyte and stretching during isovolumic relaxation, GWW represents systolic stretching of cardiomyocyte and shortening during isovolumic relaxation, and GWE is calculated from GCW divided with sum of GCW and GWW [18]. The protocol for myocardial work assessment included the utilization of Automated Functional Imaging (AFI) and its myocardial work module on Echo PAC 202 PC software. The echocardiographer entered the blood pressure values (systolic cuff pressure measurement) and valvular event times (time from mitral valve closure to mitral valve opening) to the predefined areas within the software. These data were utilized by the software to compute the global myocardial work (average of all segmental values) but also to determine the segmental work as strain–pressure loops.

Left ventricular strain imaging evaluates the global and segmental myocardial deformation, which is related to cardiac contractility and fibrosis [19]. There are 3 types of myocardial strain, including the longitudinal, radial, and circumferential strain. All strain types are dependent on cardiomyocyte shortening and represent left ventricular systolic function [5]. The left ventricular longitudinal strain was performed using the AFI on Echo PAC 202 PC software. After obtaining three standard apical views (apical long-axis, four and two chamber), the apical long-axis view was used to define end-systole by closure of the aortic valve. Subsequently, three index points of mitral annulus and left ventricular apex were defined in each of the views at end-systole. Endocardium borders, i.e., speckles within the endocardium, are automatically traced and tracked during the entire cardiac cycle. The software then automatically defines changes in length of the tracked speckle, where lengthening (during diastole) is expressed as positive value and shortening (during systole) is expressed as negative value. The difference between the end-diastolic and end-systolic value is divided with the original reference length giving the value of strain expressed in percentage. This is performed for each region of the myocardium and the software at the end provides a bullseye of the left ventricle giving us values of LS for each of the segments and also the average value, known as global longitudinal strain. In case the automatic endocardial border tracking is not correct, the echocardiographer can readjust the region of interest and recalculate the values.

The right ventricle was evaluated using the standard 2-dimensional and 4-dimensional probe standard (parasternal long-axis view; apical 4-chamber view) and modified apical projections for the right ventricle. Furthermore, the 4D probe was used to obtain multi-slice images of the right ventricle that were then used for volumetric measurements.

### 2.7. Outcomes

The main outcome of this study was the comparison of echocardiographic measurements about hemodialysis-to-hemodiafiltration transfer (baseline vs. 3-month follow-up values). The secondary outcome included the comparison of baseline echocardiographic measurements to reference values from the general population (median values vs. reference values). Other outcomes were laboratory parameters and the efficacy of dialysis regarding the hemodialysis-to-hemodiafiltration transfer (baseline vs. 3-month follow-up values).

### 2.8. Statistical Analysis

Statistical data analysis was carried out using a Statistical Package for the Social Sciences (SPSS) software (IBM Corp, NY, USA; version 20). Continuous data were presented as median (interquartile range [IQR]), while categorical variables were expressed as numbers (percentages). This analysis used non-parametric tests, such as the Mann–Whitney test, as they do not assume a specific distribution of the data, making it more robust and reliable in analyses with limited sample sizes. The baseline echocardiographic measurements were compared to the general population by evaluating the deviation of the median from the established reference ranges for each echocardiographic parameter, as defined by the Normal Reference Ranges for Echocardiography (NORRE) of the European Association of Cardiovascular Imaging (EACVI) and other relevant studies [16,20,21,22,23,24,25]. Additional data analysis was performed to determine the 95% confidence intervals (95%CI) and compare them with the established reference ranges for the main left ventricular myocardial work and longitudinal strain parameters. To test the within-subjects differences in echocardiographic measurements over the follow-up, we used the Mann–Whitney U test. A two-sided *p*-value of <0.05 was considered significant.

Sample size analysis was based on the values of myocardial global work index, suggesting that a sample size of 49 patients will allow sufficient power (β = 0.2) to detect difference from the reference population (α = 0.05). However, due to limited recruitment that was dependent on the population- and institution-related factors, it was not possible to meet this sample. Post hoc power analysis suggests 67.6% power of the present analysis.

## 3. Results

### 3.1. Baseline Patient Characteristics

The enrolled patients were mostly middle-aged (median of 62 years), with a higher proportion of male patients (58.3%). The median duration of hemodialysis treatment was 4 years, while most patients were dialysed using the arterio-venous fistula (66.7%). The patients suffered from a high comorbidity burden, including arterial hypertension (86.1%), diabetes mellitus (25.0%), and peripheral artery disease (30.5%) (Supplementary Table S1).

### 3.2. Comparison of Baseline Echocardiographic Measurements with Reference Values

**Figure 1:**
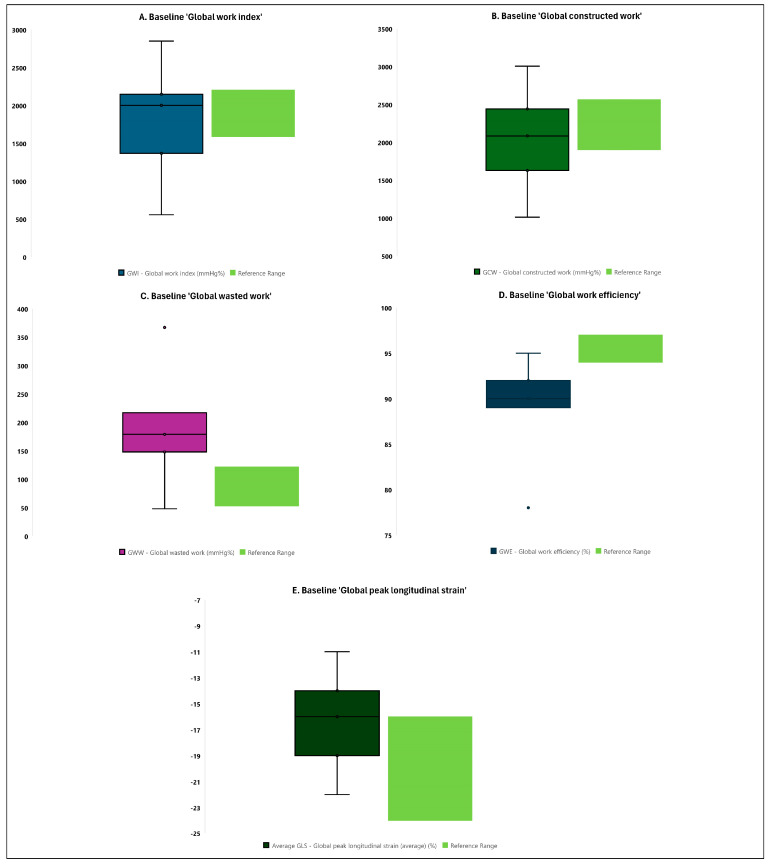
Comparison of baseline contemporary echocardiographic measures of left ventricular myocardial work and longitudinal strain to reference values from the general population (boxes and whiskers plots). Data are presented as boxes and whiskers (median (interquartile range) and minimum/maximum).

When analyzing the left ventricular myocardial work and longitudinal strain parameters, the majority of patients with ESRD showed a deviation from the reference range for global wasted work (median of 179.0 vs. 53.0–122.2 mmHg% as reference range), global work efficiency (median of 90.0 vs. 94.0–97.0% as reference range), and average global peak longitudinal strain (median of −16.0 vs. −24.0–(−16.0)% as reference range) (Figure 1 and Supplementary Table S2). There was no deviation for the global work index and global constructed work (Figure 1 and Supplementary Table S2).

To further evaluate the sample data distribution, the 95%CI were determined for the main left ventricular myocardial work and longitudinal strain parameters. This additional analysis suggested consistent findings suggesting that the true measured values of global wasted work and global work efficiency in ESRD patients will, 95% of the time, be outside of the reference ranges (global wasted work: 95%CI of 152.0–223.2 vs. 53.0–122.2 mmHg% as reference range; global work efficiency: 95%CI of 87.2–91.6 vs. 94.0–97.0% as reference range). This was not confirmed for other parameters such as the global work index, global constructed work, and global longitudinal strain (Supplementary Figure S2).

When evaluating the left atrial and ventricular 2D volumes, linear dimensions, and ejection fraction parameters, patients with ESRD showed a deviation from reference range for the left atrial diameter (median of 39.0 vs. 29.3–37.9 mm as reference range), left ventricular end-diastolic indexed volume (median of 114.3 vs. 40.0–62.8 mL/m^2^ as reference range), left ventricular end-systolic indexed volume (median of 55.7 vs. 13.4–23.8 mL/m^2^ as reference range), left ventricular ejection fraction (median of 57.0 vs. 59.0–68.8% as reference range), left ventricular mass (median of 240.4 vs. 89.4–164.2 g as reference range), and indexed left ventricular mass (median of 122.9 vs. 52.4–87.4 g/m^2^ as reference range) (Table 1).

When analyzes the right ventricular size and function parameters, patients with ESRD showed a deviation from the reference range for tricuspid annular plane systolic excursion (median of 12.2 vs. ≥17.0 mm as reference range) and right ventricular fractional area change (median of 36.6 vs. 41.3–58.1% as reference range) (Table 2).

When analyzing the left ventricular diastolic function and Doppler parameters, patients with ESRD showed a deviation from the reference range for transmitral E wave velocity (median of 80.0 vs. <50.0 cm/s as reference range) and mitral valve deceleration time (median of 291.0 vs. 160.0–220.0 ms as reference range) (Table 3).

### 3.3. Echocardiographic Parameters Following the Hemodialysis-to-Hemodiafiltration Transfer

Following the hemodialysis-to-hemodiafiltration transfer, there was no statistically significant change in the left ventricular myocardial work and longitudinal strain parameters, except in global wasted work that increased (179.0 vs. 233.5 mmHg%, *p* = 0.037), aortic valve opening time that decreased (94.0 vs. 70.0 ms, *p* = 0.038), and mid-inferior peak systolic longitudinal strain that improved (−17.0 vs. −18.7%, *p* = 0.016) at 3-month follow-up (Table 4).

Furthermore, there was no statistically significant change in the left atrial and ventricular 2D volumes, linear dimensions, and ejection fraction parameters, except in interventricular septal diameter that decreased (10.0 vs. 9.0 mm, *p* = 0.013), left ventricular posterior wall diastolic diameter that decreased (11.0 vs. 9.0 mm, *p* = 0.004), and left ventricular posterior wall systolic diameter that decreased (14.0 vs. 13.0 mm, *p* = 0.028) at 3-month follow-up (Table 5).

When evaluating the left ventricular diastolic function and Doppler parameters, there was no statistically significant change following the hemodialysis-to-hemodiafiltration transfer (Table 6).

Finally, there was no statistically significant change in the right ventricular size and function parameters, except in the right ventricular diastolic diameter at mid-ventricle that increased (26.7 vs. 28.9 mm, *p* = 0.042) following the hemodialysis-to-hemodiafiltration transfer (Table 7).

### 3.4. Evaluation of the Laboratory Parameters and the Efficacy of Dialysis during the 3-Month Follow-Up

There was no statistically significant difference in the main laboratory parameters following the hemodialysis-to-hemodiafiltration transfer, except in hemoglobin levels that decreased at 3-month follow-up (119.0 vs. 109.0 g/L, *p* = 0.009) (Supplementary Table S3).

Following the hemodialysis-to-hemodiafiltration transfer, there was a significant decrease in the levels of β2 microglobulin (46.8 vs. 9.6 mg/L, *p* < 0.001) and a statistically significant increase in the Kt/V ratio (1.2 vs. 1.4, *p* = 0.024), indicating a higher efficacy of dialysis treatment with hemodiafiltration (Supplementary Table S4).

## 4. Discussion

This study provides important data about contemporary echocardiographic measures in patients with ESRD undergoing hemodialysis-to-hemodiafiltration transfer. To the best of our knowledge, there are no studies evaluating the myocardial work and strain in patients undergoing hemodialysis-to-hemodiafiltration transfer. Several prior studies compared the conventional echocardiographic measures between patients receiving conventional hemodialysis and those receiving on-line hemodiafiltration [7,26,27,28,29]. Furthermore, few studies evaluated contemporary echocardiographic measures solely in patients receiving conventional hemodialysis [30,31,32,33], but not concerning transfer to hemodiafiltration. The comprehensive assessment of contemporary echocardiographic parameters in ESRD patients, both at baseline and during the transition between dialysis techniques, could contribute to our understanding of cardiac dynamics in this population. An additional purpose of this underpowered study is its hypothesis-generating potential for future research on advanced cardiovascular imaging in patients with terminal renal disease. It may also stimulate further research and the development of novel non-invasive cardiovascular diagnostic and therapeutic modalities in this population.

There are several main findings of this study. First, patients with ESRD showed baseline deviations of global wasted work, global work efficiency, and average global peak longitudinal strain, suggesting an impaired myocardial work and strain function compared to the general population. Second, patients with ESRD exhibited significantly higher left atrial diameters, left ventricular volumes, and left ventricular mass compared to the general population, which is consistent with chronic volume/pressure overloading. Third, the raw measures of left ventricular (ejection fraction) and right ventricular systolic function (tricuspid annular plane systolic excursion and fractional area change) were impaired in ESRD patients suggesting the biventricular affection in this population. Finally, following the hemodialysis-to-hemodiafiltration transfer, there was no significant change in most contemporary and conventional echocardiographic measures, except for an increase in global wasted work.

Hemodiafiltration has been associated with better cardiovascular outcomes compared to hemodialysis [1,7,8,34], but objective methods of cardiovascular assessment are lacking. There is evidence that hemodiafiltration is associated with a better hemodynamic balance with less intradialytic hypotension, better blood pressure control, and more stable endothelial function [1]. Additionally, a more effective removal of middle-weight molecules, such as β2-microglobulin, may mitigate myocardial hypertrophy in this population. Takayama et al. published a high proportion of amyloid fibrils in the myocardium of long-term chronic maintenance hemodialysis patients [35]. Hypothetically, a better removal of other middle-weight molecules such as cytokines, advanced glycation end products, pentraxin-3, visfatin, adiponectin, leptin, and fibroblast growth factors could be associated with slower progressive atherosclerosis and valve calcification and therefore can slow down the progression of coronary artery disease and valve disease [9].

The speckle-tracking-derived global longitudinal strain emerged as an important contemporary echocardiographic parameter for the early detection of left ventricular dysfunction [36]. Several studies showed its relevance in detecting subclinical myocardial dysfunction amongst patients receiving conventional hemodialysis, particularly in those with a preserved ejection fraction [5,15,30,31,37]. These studies suggest that global longitudinal strain outperforms ejection fraction in predicting both all-cause and cardiovascular mortality, emphasizing its importance in this specific patient group [14]. Previous studies have also shown an association of impaired global longitudinal strain with impaired prognosis in this population [12,13]. The present study showed lower values of global longitudinal strain in hemodialysis patients, when compared to established reference intervals, suggesting the presence of left ventricular dysfunction in this population. This is consistent with previous studies comparing ESRD patients and the general population [30,31,33,38]. However, due to the specific characteristics of ESRD patients and their unique pathophysiology, there are potential biases in using general population values as a reference. Specifically, ESRD patients have inherently increased cardiac loading conditions and frequently exhibit humoral disbalance that may lead to microvascular dysfunction, cardiomyocyte impairments, and electrolyte-related channelopathies. Therefore, it is unclear if ESRD patients require different cut-off values for contemporary echocardiographic measures, and careful interpretation of these findings is mandatory.

Another contemporary echocardiographic measure of significance is myocardial work, which has emerged as a novel and sensitive parameter for evaluating systolic function. Despite not being widely incorporated into everyday clinical practice, myocardial work has the potential for broader utilization due to its ability to consider dynamic cardiac contraction in various loading circumstances and afterload-dependent restrictions [17]. There is growing interest in exploring the applicability of myocardial work in assessing left ventricular function in patients undergoing chronic maintenance hemodialysis [39]. The present study revealed that hemodialysis patients exhibit a significantly impaired ‘myocardial work’ function, including a decreased GWE and increased GWW, compared to established reference intervals. This is consistent with a recent study showing significantly reduced GWE in patients undergoing chronic maintenance hemodialysis, but there was no significant difference in left ventricular ejection fraction [40], which is contrary to the findings from this analysis. This disagreement may be associated with a difference in hemodialysis efficiency, or due to differences in the reference population (age- and sex-matched controls subjects vs. reference values from the general population) [40]. Further research in this area is therefore warranted to establish the potential clinical applications and implications of myocardial work measurements in the ESRD population.

A crucial aspect of the study involved comparing contemporary echocardiographic measurements with subsequent control findings post-hemodialysis-to-hemodiafiltration transfer. A transfer to hemodiafiltration may be associated with positive cardiovascular effects [1,7,8,9,10], and there are several potential mechanistic hemodynamic effects of hemodiafiltration versus hemodialysis that could mediate differences in the myocardial work indices [29,32,33]. Firstly, hemodiafiltration should include more gradual and controlled fluid removal due to its combination of diffusion and convection, thereby avoiding significant oscillations of autonomous nervous system and peripheral vascular resistance (afterload) [29]. Secondly, controlled fluid removal with hemodiafiltration may prevent sudden shifts in intravascular volume, thereby maintaining a more stable preload. Thirdly, hemodiafiltration may have more favourable effects on the myocardial work indices by avoiding sudden changes in cardiac loading conditions, which translate to increased myocardial oxygen demand [32,33]. Nevertheless, this analysis did not show significant difference in most parameters of the global longitudinal strain and myocardial work, except for the mid-inferior peak systolic longitudinal strain that improved following the transfer, coupled with an increase in global wasted work. The observed beneficial changes in segmental longitudinal strain could represent an early echocardiographic sign of hemodynamic effects of hemodiafiltration. However, the unchanged global longitudinal strain and paradoxically increased global wasted work may reflect more complex associations in vivo. Firstly, global longitudinal strain is sensitive to an increased wall stress [31], which is not necessarily resolved with hemodiafiltration. Despite theoretically more effective and gradual volume reduction with hemodiafiltration, the variability of sympathetic activation, and compensatory increase in arterial afterload is unpredictable, thereby potentially promoting an increase in global wasted work. Secondly, this study did not account for the measures of cardiac electromechanical synchrony such as the presence of bundle branch blocks, which may affect the global wasted work [20] and may bias the observed findings. Fourthly, cardiac remodelling may require more time than the scheduled post-transfer follow-up. Finally, the underpowered sample size of the study could hamper the differentiation of any statistical difference between the time points. Future powered studies are therefore needed to detect the underlying trends and mechanisms of the interaction between hemodiafiltration and myocardial work/strain in ESRD patients. The current body of literature does not contain any study investigating contemporary echocardiographic measures concerning hemodialysis-to-hemodiafiltration transfer.

The commonly utilized conventional measures of left and right ventricular systolic function were found to be impaired in hemodialysis patients in this study, a finding consistent with previous research [32,41]. This indicates a biventricular dysfunction within this patient population, likely attributable to a combination of volume and pressure overload, along with latent ischemia caused by microcirculatory dysfunction resulting from hemodynamic oscillations in myocardial perfusion during hemodialysis sessions [5]. Additionally, the direct impact of uremia may further contribute to the observed myocardial impairment. This study also observed a high proportion of left ventricular hypertrophy, volume enlargement, and increased mass—all recognized as robust predictors of cardiovascular mortality. These conditions, often associated with both high-volume overload and pressure overload, have been consistently reported in prior studies [42,43]. These findings underscore the intricate relationship between hemodialysis and adverse cardiac remodelling, emphasizing the need for targeted interventions to mitigate the heightened cardiovascular risks in this patient cohort.

Several studies investigated the conventional echocardiographic parameters, such as the left ventricular ejection fraction and mass, in distinct patient groups receiving different hemodialysis types [7,26,28,29]. Studies comparing the hemodialysis and hemodiafiltration cohorts revealed a significant increase in ejection fraction following the treatment initiation in the hemodiafiltration cohort, but not in those treated with hemodialysis [28,44]. Similarly, the beneficial effects of hemodiafiltration on the regression of left ventricular mass were previously reported [7,45]. However, the available literature reports are inconsistent, with meta-analysis suggesting no significant association of left ventricular ejection fraction and mass between patients receiving hemodialysis and hemodiafiltration treatment [26,29]. This is consistent with the present study, showing no difference in left ventricular ejection fraction and mass following the hemodialysis-to-hemodiafiltration transfer in a single patient group. Therefore, the pathophysiology of left ventricular hypertrophy and impairment may be multifactorial and not necessarily related to the treatment received.

This study has several noteworthy limitations that merit emphasis. Firstly, the limited sample size poses a constraint on the power of the analysis and the generalizability of the findings. Secondly, the inclusion of single time point during the follow-up within a relatively short period hampers the ability to monitor the dynamics of changes and draw inferences about long-term follow-up data. Thirdly, it is crucial to acknowledge the presence of confounding variables, particularly concerning the possibility of subclinical cardiovascular events that could impact myocardial function, thereby influencing echocardiographic findings. Fourthly, it is essential to recognize that echocardiography, being an operator-dependent technique, may introduce potential deviations in measurements. Specifically, despite eliminating the bias of inter-observer variability by focusing on only one experienced echocardiographer, this analysis contained a residual bias of intra-observer variability and bias of within-subject variability. Fifthly, a comparison with sex- and age-matched controls without renal disease would be more appropriate than a comparison with reference values. Also, parallel evaluation of echocardiographic changes in with patients continuing standard haemodialysis may add some additional benefit to the study. Furthermore, this study shares inherent limitations common to observational research. Finally, the parameters of volume status, such as bioimpedance, lung ultrasound, inferior vena cava diameter, interdialytic weight changes, and ultrafiltration per dialysis session, were not monitored for this study. Consequently, the potential effects of these parameters on echocardiographic measurements could not be thoroughly evaluated, introducing a limitation in the comprehensiveness and depth of the analysis.

There are several clinical and scientific implications of this study. Firstly, it provides novel data suggesting impaired contemporary echocardiographic measures such as global longitudinal strain and myocardial work in hemodialysis patients. Having in mind an association of these parameters with cardiovascular prognosis [11,12,13,14], this study may urge clinicians for routine and early advanced echocardiographic referral in this population. An early detection of subtle cardiac impairment by advanced echocardiography may help in risk stratification and the timely detection of patients at risk. This information may translate into closer clinical follow-up, stronger preventive measures, stringent lipid lowering, and modifications of renal replacement therapy. Increased clinician’s awareness may improve the cardiovascular risk reduction by strict blood pressure control, smoking cessation programmes, or diet modifications. In addition, early prescription of cardioprotective medications, such as sodium–glucose cotransporter inhibitors, which are currently not well investigated in ESRD patients, may gain more attention. Secondly, these findings support the need for routine multidisciplinary team assessment in hemodialysis patients to meet the rising demands of the disease burden. Regular cardiologic counselling for ESRD patients could therefore become the standard of care, particularly in those with subtle echocardiographic impairments, to timely prevent adverse events [11,12,13,14]. Finally, this underpowered study has a hypothesis-generating purpose by setting a background for further research in this insufficiently researched area. Future powered studies on this topic may lead to the development of ESRD-specific reference ranges for myocardial work and strain, while future risk stratification tools in ESRD population could incorporate advanced echocardiographic parameters. Hemodiafiltration warrants more scientific attention to clearly delineate whether it truly represents a more favourable dialysis modality from cardiovascular perspective. The development of targeted interventions and monitoring strategies to address the unique cardiovascular challenges faced by individuals with ESRD is prioritized.

## 5. Conclusions

In conclusion, this study highlights baseline deviations in global wasted work, global work efficiency, and average global peak longitudinal strain among patients with ESRD undergoing hemodialysis, indicative of impaired myocardial work and strain function compared to the general population. Notably, the transition from hemodialysis to hemodiafiltration showed no significant changes in most contemporary echocardiographic measures. These findings warrant further exploration through additional sufficiently powered prospective studies in the field.

## Figures and Tables

**Table 1:** Comparison of baseline echocardiographic measures of the left atrial and ventricular 2D volumes, linear dimensions, and ejection fraction to reference values from the general population.

Echocardiographic Parameters	Study Sample (Baseline—Hemodialysis Treatment)	Reference Values
LAd—Left atrial diameter (mm)	39.0 (35.0–42.0) *	29.3–37.9
LAVi—Left atrial volume indexed (mL/m^2^)	26.0 (19.3–28.15)	18.0–31.6
LVEDV BiP—Left ventricular end-diastolic volume [bi-plane] (mL)	92.5 (67.0–106.0)	68.0–117.6
LVESV BiP—Left ventricular end-systolic volume [bi-plane] (mL)	41.2 (25.0–48.0)	22.8–44.6
LVEF BiP—Left ventricular ejection fraction [bi-plane] (%)	57.0 (53.0–63.0)	59.0–68.8
LVEDVi—Left ventricular end-diastolic volume indexed (mL/m^2^)	114.3 (84.0–125.0) *	40.0–62.8
LVESVi—Left ventricular end-systolic volume indexed (mL/m^2^)	55.7 (37.0–69.0) *	13.4–23.8
IVSd—Interventricular septal wall thickness (mm)	10.0 (9.0–11.0)	7.0–10.2
LVPWd—Left ventricular posterior wall thickness (mm)	11.0 (10.0–11.0)	7.3–10.3
LVIDd—Left ventricular internal end-diastolic diameter (mm)	51.0 (46.0–54.0)	39.5–49.1
LVIDs—Left ventricular internal end-systolic diameter (mm)	33.0 (26.0–38.0)	25.2–34.6
LVM—Left ventricular mass (g)	240.4 (185.1–280.4) *	89.4–164.2
LVMi—Left ventricular mass indexed (g/m^2^)	122.9 (90.6–153.9)	52.4–87.4

Data are expressed as median (interquartile range). * Outside of the reference range. Abbreviations: none.

**Table 2:** Comparison of baseline echocardiographic measures of the right ventricular size and function parameters to reference values from the general population.

Echocardiographic Parameters	Study Sample (Baseline—Hemodialysis Treatment)	Reference Values
RVEDV—Right ventricular end-diastolic volume (mL)	65.0 (53.0–66.0)	65.0–107.0
RVESV—Right ventricular end-systolic volume (mL)	34.4 (29.0–40.0)	18.0–40.0
3D RVEF (%)—3D right ventricular ejection fraction (%)	45.9 (41.8–51.2) *	>45.0
RV Dd base—Right ventricular diastolic diameter—base (mm)	37.1 (32.0–40.0)	28.7–39.7
RV Dd mid—Right ventricular diastolic diameter—mid (mm)	26.7 (23.0–28.0)	22.5–33.5
RV Ld—Right ventricular longitudinal diameter (mm)	63.1 (59.0–67.0)	59.8–75.8
TAPSE—Tricuspid annular plane systolic excursion (mm)	12.2 (9.0–16.0) *	≥17.0
RV FAC—Right ventricular fractional area change (%)	36.6 (31.2–36.6) *	41.3–58.1

Data are expressed as median (interquartile range). * Not within the reference range. Abbreviations: none.

**Table 3:** Comparison of baseline echocardiographic measures of diastolic function and Doppler parameters to reference values from the general population.

Echocardiographic Parameters	Study Sample (Baseline—Hemodialysis Treatment)	Reference Values
MV E vel—Transmitral E wave velocity (cm/s)	80.0 (70.0–100.0) *	<50.0
MV DecT—Mitral valve deceleration time (ms)	291.0 (257.0–356.0) *	160.0–220.0
MV E/A Ratio—Mitral valve E/A Ratio	0.8 (0.6–1.0)	0.8–2.0
e’ Sept—Septal annular e′ velocity (cm/s)	9.8 (5.2–11.3)	>7.0
e’ lat—Lateral annular e′ velocity (cm/s)	10.1 (8.5–11.8)	>10.0
E/e’ average ratio	10.8 (8.7–12.6)	<14.0
PV AccT—Pulmonic valve acceleration time (ms)	155.0 (135.0–166.0)	>120.0
TV S’—Peak systolic tissue velocity (S’ wave) at the tricuspid annulus (cm/s)	10.0 (9.3–11.2)	>9.5

Data are expressed as median (interquartile range). * Not within the reference range. Abbreviations: none.

**Table 4:** Comparison of contemporary echocardiographic measures of left ventricular myocardial work and longitudinal strain following the hemodialysis-to-hemodiafiltration transfer.

Echocardiographic Parameters	Baseline (Hemodialysis Treatment)	3-Month Follow-Up (Hemodiafiltration Treatment)	*p*-Value
GWI—Global work index (mmHg%)	2001.0 (1368.0–2148.0)	1897.5 (1669.0–2396.0)	0.637
GCW—Global constructed work (mmHg%)	2084.5 (1628.0–2440.0)	1939.5 (1793.0–2387.0)	0.886
GWW—Global wasted work (mmHg%)	179.0 (148.0–217.0)	233.5 (159.0–315.0)	0.037
GWE—Global work efficiency (%)	90.0 (89.0–92.0)	89.0 (87.0–91.0)	0.250
AVO—Aortic valve opening (ms)	94.0 (63.0–115.7)	70.0 (50.0–93.0)	0.038
AVC—Aortic valve closure (ms)	361.0 (298.0–384.0)	347.0 (330.0–371.0)	0.422
MVO—Mitral valve opening (ms)	449.7 (411.0–483.0)	453.6 (424.0–471.0)	0.817
MVC—Mitral valve closure (ms)	329.0 (19.0–674.0)	706.0 (53.0–849.0)	0.063
Longitudinal peak strain dispersion (ms)	64.0 (51.0–70.0)	58.1 (51.0–65.0)	0.385
APLAX GLS—Global peak longitudinal strain [APLAX] (%)	−16.0 (−18.0–[−14.0])	−17.1 (−18.0–[−16.0])	0.293
A4C GSL (A4C)—Global peak longitudinal strain [A4C] (%)	−16.0 (−19.0–[−14.0])	−17.0 (−18.0–[−16.0])	0.376
A2C GLS (A2C)—Global peak longitudinal strain [A2C] (%)	−18.0 (−20.0–[−14.0])	−18.2 (−20.0–[−17.0])	0.440
Average GLS—Global peak longitudinal strain (average) (%)	−16.0 (−19.0–[−14.0])	−17.5 (−19.0–[−17.0])	0.258
BA PSSL Full (%)—Basal anterior peak systolic longitudinal strain (%)	−13.8 (−18.0–[−9.0])	−15.0 (−16.0–[−12.0])	0.516
BI PSSL Full (%)—Basal inferior peak systolic longitudinal strain (%)	−15.0 (−17.0–[−10.0])	−15.8 (−18.0–[−14.0])	0.117
MA PSSL Full (%)—Mid-anterior peak systolic longitudinal strain (%)	−15.0 (−19.0–[−9.0])	−15.0 (−18.0–[−13.0])	0.826
MI PSSL Full (%)—Mid-inferior peak systolic longitudinal strain (%)	−17.0 (−19.0–[–11.0])	−18.7 (−20.0–[−18.0])	0.016
AA PSSL Full (%)—Apical anterior peak systolic longitudinal strain (%)	−21.0 (−29.0–[−18.0])	−21.0 (−24.0–[−17.0])	0.590
AI PSSL Full (%)—Apical inferior peak systolic longitudinal strain (%)	−22.0 (−32.0–[−18.0])	−25.5 (−31.0–[−23.0])	0.379
BAS PSSL Full (%)—Basal anteroseptal peak systolic longitudinal strain (%)	−12.0 (−14.0–[−8.0])	−13.0 (−16.0–−10.0])	0.107
BP PSSL Full (%)—Basal posterior peak systolic longitudinal strain (%)	−14.0 (−18.0–[−10.0])	−15.3 (−17.0–[−12.0])	0.467
MAS PSSL Full (%)—Mid-anteroseptal peak systolic longitudinal strain (%)	−20.0 (−23.0–[−15.0])	−20.1 (−23.0–[−18.0])	0.441
MP PSSL Full (%)—Mid-posterior peak systolic longitudinal strain (%)	−17.0 (−19.0–[−13.0])	−16.1 (−19.0–[−12.0])	0.930
AS PSSL Full (%)—Apico-anteroseptal peak systolic longitudinal strain (%)	−24.0 (−29.0–−14.0])	−23.0 (−27.0–[−21.0])	0.733
AP PSSL Full (%)—Apical posterior peak systolic longitudinal strain (%)	−20.0 (−28.0–[−14.0])	−18.3 (−20.0–[−16.0])	0.590
BS PSSL Full (%)—Basal septal peak systolic longitudinal strain (%)	−11.0 (−15.0–[−6.0])	−11.7 (−13.0–[−10.0])	0.567
BL PSSL Full (%)—Basal lateral peak systolic longitudinal strain (%)	−13.0 (−17.0–[−11.0])	−14.0 (−17.0–[−12.0])	0.800
MS PSSL Full (%)—Mid-septal peak systolic longitudinal strain (%)	−18.0 (−21.0–[−14.0])	−19.0 (−21.0–[−17.0])	0.454
ML PSSL Full (%)—Mid-lateral peak systolic longitudinal strain (%)	−17.0 (−19.0–[−12.0])	−16.6 (−19.0–[−14.0])	0.692
AS PSSL Full (%)—Apical septal peak systolic longitudinal strain (%)	−23.0 (−29.0–[−16.0])	−23.0 (−26.0–[−21.0])	0.912
AL PSSL Full (%)—Apical lateral peak systolic longitudinal strain (%)	−21.0 (−27.0–[−12.0])	−18.5 (−23.0–[−14.0])	0.545

Data are expressed as median (interquartile range) and compared using the Mann–Whitney U test. Abbreviations: none.

**Table 5:** Comparison of contemporary echocardiographic measures of left atrial and ventricular 2D volumes, linear dimensions, and ejection fraction following the hemodialysis-to-hemodiafiltration transfer.

Echocardiographic Parameters	Baseline (Hemodialysis Treatment)	3-Month Follow-Up (Hemodiafiltration Treatment)	*p*-Value
Left atrium			
LALs A2C—Left atrial length—systolic [A2C] (mm)	53.0 (41.0–57.0)	54.0 (48.0–59.0)	0.190
LAESV A-L A2C—Left atrial end-systolic volume, area-length [A2C] (mL)	49.0 (32.0–68.0)	52.0 (45.0–71.0)	0.333
LAESV MOD A2C—Left atrial end-systolic volume, method of discs [A2C] (mL)	47.0 (31.0–67.0)	54.0 (42.0–68.0)	0.291
LALs A4C—Left atrial length—systolic [A4C] (mm)	56.0 (50.0–62.0)	56.0 (54.0–62.0)	0.620
LAESV A-L A4C—Left atrial end-systolic volume, area-length [A4C] (mL)	44.5 (35.0–60.0)	52.0 (43.0–57.0)	0.692
LAESV MOD A4C—Left atrial end-systolic volume, method of discs [A4C] (mL)	44.0 (33.0–60.0)	51.0 (40.0–58.0)	0.590
LAESV AL—Left atrial end-systolic volume, area-length [derived] (mL)	51.0 (39.0–65.0)	55.0 (45.0–61.0)	0.409
LAVi—Left atrial volume indexed (mL/m^2^)	26.0 (19.3–28.15)	28.1 (23.5–30.6)	0.214
Left ventricle			
LVVED 4Ch—Left ventricular end-diastolic volume [4Ch] (mL)	76.0 (59.0–103.0)	77.3 (68.0–84.0)	0.826
LVVES 4Ch—Left ventricular end-systolic volume [4Ch] (mL)	32.0 (20.0–44.0)	36.4 (30.0–45.0)	0.575
LVEF 4Ch—Left ventricular ejection fraction [4Ch] (%)	57.2 (50.0–66.0)	53.1 (51.0–56.0)	0.153
LVSV 4Ch—Left ventricular stroke volume [4Ch] (mL)	42.0 (39.0–53.0)	40.8 (28.0–47.0)	0.190
LVCO 4Ch—Left ventricular cardiac output [4Ch] (L/min)	3.6 (2.6–4.1)	(2.8–3.4)	0.053
LVLs 4Ch—Left ventricular length in systole [4Ch] (mm)	61.0 (55.0–66.0)	61.0 (58.0–67.0)	0.523
LVLd 4Ch—Left ventricular length in diastole [4Ch] (mm)	72.0 (67.0–77.0)	73.0 (72.0–77.0)	0.461
LVVED 2Ch—Left ventricular end-diastolic volume [2Ch] (mL)	89.0 (56.0–107.0)	85.0 (74.0–90.0)	0.982
LVVES 2Ch—Left ventricular end-systolic volume [2Ch] (mL)	32.0 (24.0–48.0)	35.3 (27.0–40.0)	0.886
LVEF 2Ch—Left ventricular ejection fraction [2Ch] (%)	57.0 (54.0–60.0)	60.2 (57.0–52.0)	0.088
LVSV 2Ch—Left ventricular stroke volume [2Ch] (mL)	47.0 (35.0–61.0)	51.0 (42.0–55.0)	0.410
LVCO 2Ch—Left ventricular cardiac output [2Ch] (L/min)	3.4 (2.5–4.3)	3.5 (3.1–3.8)	0.676
LVLs 2Ch—Left ventricular length in systole [2Ch] (mm)	6.3 (5.7–6.9)	6.4 (5.9–6.7)	0.582
LVLs 2Ch—Left ventricular length in diastole [2Ch] (mm)	7.4 (6.6–8.0)	7.7 (7.1–8.1)	0.317
LVVED BiP—Left ventricular end-diastolic volume [biplane] (mL)	92.5 (67.0–106.0)	82.8 (72.0–97.0)	0.367
LVVES BiP—Left ventricular end-systolic volume [biplane] (mL)	41.2 (25.0–48.0)	36.6 (30.0–39.0)	0.488
LVEF BiP -Left ventricular ejection fraction [biplane] (%)	57.0 (53.0–63.0)	56.5 (55.0–60.0)	0.644
LVSV BiP—Left ventricular stroke volume [biplane] (mL)	49.0 (38.0–58.0)	46.2 (42.0–29.0)	0.530
LVCO BiP—Left ventricular cardiac output [biplane] (L/min)	3.7 (3.0–4.0)	3.2 (3.0–3.4)	0.052
LVVED 4D—Left ventricular end-diastolic volume [4-dimensional] (mL)	114.3 (84.0 –125.0)	103.6 (73.0–122.0)	0.404
LVVES 4D—Left ventricular end-systolic volume [4-dimensional] (mL)	55.7 (37.0–69.0)	46.9 (34.0–54.0)	0.126
CO 4D—Cardiac output [4-dimensional] (L/min)	4.0 (3.2–4.5)	3.7 (2.9–4.6)	0.344
Spl—Sphericity index	0.4 (0.3–0.5)	0.4 (0.3–0.5)	0.455
IVSd—Interventricular septal thickness in diastole (mm)	10.0 (9.0–11.0)	9.0 (8.0–10.0)	0.013
LVIDd—Left ventricular internal end-diastolic diameter (mm)	51.0 (46.0–54.0)	51.0 (48.0–55.0)	0.860
LVPWd—Left ventricular posterior wall thickness in diastole (mm)	11.0 (10.0–11.0)	9.0 (8.0–10.0)	0.004
IVSs—Interventricular septal thickness in systole (mm)	13.0 (11.0–14.0)	12.0 (11.0–13.0)	0.062
LVIDs—Left ventricular internal end-systolic diameter (mm)	33.0 (26.0–38.0)	33.0 (31.0–36.0)	0.461
LVPWs—Left ventricular posterior wall thickness in systole (mm)	14.0 (13.0–15.0)	13.0 (12.0–14.0)	0.028
EDV Teich—Left ventricular end-diastolic volume [Teicholz] (mL)	127.1 (96.0–140.0)	127.0 (105.0–147.0)	0.904
ESV Teich—Left ventricular end-systolic volume [Teicholz] (mL)	46.9 (24.0–61.0)	47.0 (38.0–54.0)	0.538
LVEF Teich—Left ventricular ejection fraction [Teicholz] (%)	64.6 (59.0–73.0)	63.1 (61.0–66.0)	0.553
%FS—Left ventricular fractional shortening (%)	36.0 (32.0–42.0)	35.0 (33.0–37.0)	0.434
LVSV Teich—Left ventricular stroke volume [Teicholz] (mL)	80.0 (57.0–107.0)	79.0 (68.0–93.0)	0.965
LVd Mass—Left ventricular mass (g)	240.4 (185.1–280.4)	211.4 (165.1–258.0)	0.150
LVMi—Left ventricular mass indexed (g/m^2^)	122.9 (90.6–153.9)	103.6 (81.6–155.5)	0.123
Ao diam—Aortic diameter (mm)	33.0 (30.0–36.0)	32.0 (30.0–35.0)	0.620
LA diam—Left atrial diameter (mm)	39.0 (35.0–42.0)	40.0 (37.0–43.0)	0.628
LA/Ao—Left atrium/aorta ratio	1.2 (1.1–1.2)	1.2 (1.1–1.4)	0.129

Data are expressed as median (interquartile range) and compared using the Mann–Whitney U test. Abbreviations: none.

**Table 6:** Comparison of contemporary echocardiographic measures of left ventricular diastolic function and Doppler parameters following the hemodialysis-to-hemodiafiltration transfer.

Echocardiographic Parameters	Baseline (Hemodialysis Treatment)	3-Month Follow-Up (Hemodiafiltration Treatment)	*p*-Value
MV E vel—Mitral valve E wave velocity (m/s)	0.8 (0.7–1.0)	0.9 (0.7–1.0)	0.397
MV DecT—Mitral valve deceleration time (ms)	291.0 (257.0–356.0)	306.5 (244.0–346.0)	0.660
MV Dec Slope—Mitral valve deceleration slope (m/s^2^)	2.8 (2.1–3.4)	3.3 (2.3–3.7)	0.361
MV A Vel—Mitral valve A wave velocity (m/s)	1.0 (0.8–1.1)	1.0 (0.9–1.2)	0.428
MV E/A Ratio—Mitral valve E/A wave ratio	0.8 (0.6–1.0)	0.9 (0.7–1.0)	0.397
Average septal annular e′ velocity (cm/s)	9.8 (5.2–11.3)	9.6 (5.3–10.9)	0.190
E/e’ Sept—E to e’ wave ratio at septal mitral annulus	13.4 (11.2–15.7)	13.7 (11.8–15.6)	0.982
Average lateral annular e′ velocity (cm/s)	10.1 (8.5–11.8)	9.7 (8.4–11.3)	0.525
E/e’ Lat—E to e’ wave ratio at lateral mitral annulus	8.9 (7.0–10.4)	9.3 (8.0–9.6)	0.613
E/e’ Avg—E/e’ average ratio	10.8 (8.7–12.6)	11.1 (9.7–11.3)	0.939
AV Vmax—Aortic valve maximal velocity (m/s)	1.5 (1.2–1.8)	1.6 (1.3–1.7)	0.403
AV Vmean—Aortic valve mean velocity (m/s)	1.2 (0.8–1.3)	1.1 (0.9–1.1)	0.684
AV maxPG—Aortic valve maximal pressure gradient (mmHg)	10.6 (6.7–12.6)	11.2 (7.2–11.9)	0.921
AV meanPG—Aortic valve mean pressure gradient (mmHg)	6.4 (3.1–7.0)	6.2 (4.0–6.4)	0.809
AV Env. Ti—Aortic valve envelope time (ms)	313.5 (262.0–352.0)	310.7 (304.0–343.0)	0.636
AV VTI—Aortic valve velocity-time integral (cm)	33.1 (26.5–35.3)	36.6 (30.0–36.8)	0.146
PV AccT—Pulmonic valve acceleration time (ms)	155.0 (135.0–166.0)	151.0 (138.0–156.0)	0.684
PV Acc Slope—Pulmonic valve acceleration slope (m/s^2^)	5.9 (4.8–6.9)	6.2 (5.0–6.7)	0.947
Peak systolic tissue velocity (S’ wave) at the tricuspid annulus (cm/s)	10.0 (9.3–11.2)	10.4 (9.1–12.2)	0.273

Data are expressed as median (interquartile range) and compared using the Mann–Whitney U test. Abbreviations: none.

**Table 7:** Comparison of contemporary echocardiographic measures of right ventricular size and function parameters following the hemodialysis-to-hemodiafiltration transfer.

Echocardiographic Parameters	Baseline (Hemodialysis Treatment)	3-Month Follow-Up (Hemodiafiltration Treatment)	*p*-Value
RVEDV—Right ventricular end-diastolic volume (mL)	65.0 (53.0–66.0)	59.9 (47.0–68.0)	0.361
RVESV—Right ventricular end-systolic volume (mL)	34.4 (29.0–40.0)	32.5 (29.0–35.0)	0.305
3D RVEF (%)—3D right ventricular ejection fraction (%)	45.9 (41.8–51.2)	45.9 (38.2–48.6)	0.434
RV SV—Right ventricular stroke volume (mL)	29.9 (24.0–33.0)	27.7 (21.0–32.0)	0.494
RV Dd base—Right ventricular diastolic diameter—base (mm)	37.1 (32.0–40.0)	35.6 (34.0–37.0)	0.152
RV Dd mid—Right ventricular diastolic diameter—mid (mm)	26.7 (23.0–28.0)	28.9 (27.0–31.0)	0.042
RVLd—Right ventricular longitudinal diameter (mm)	63.1 (59.0–67.0)	60.5 (58.0–61.0)	0.134
TAPSE—Tricuspid annular plane systolic excursion (mm)	12.2 (9.0–16.0)	12.4 (10.0–14.0)	0.886
RV FAC—Right ventricular fractional area change (%)	36.6 (31.2–36.6)	39.5 (31.0–42.4)	0.182

Data are expressed as median (interquartile range) and compared using the Mann–Whitney U test. Abbreviations: none.

## Data Availability

The datasets used and/or analyzed during the current study are available from the corresponding author on reasonable request.

## Supplementary Materials

The following supporting information can be downloaded at https://www.mdpi.com/article/10.3390/medicina60091537/s1, Supplementary Figure S1. Flow diagram; Supplementary Figure S2. Comparison of baseline contemporary echocardiographic measures of left ventricular myocardial work and longitudinal strain to reference values from the general population (mean and 95% confidence intervals); Supplementary Table S1. Baseline characteristics of the study sample; Supplementary Table S2. Comparison of baseline contemporary echocardiographic measures of left ventricular myocardial work and longitudinal strain to reference values from the general population; Supplementary Table S3. Baseline laboratory parameters of the study sample; Supplementary Table S4. Relevant laboratory measurements regarding the dialysis treatment: pre-dialysis and post-dialysis (at study onset and 3-month follow-up). Supplementary STROBE Statement—checklist of items that should be included in reports of observational studies.
