# Supplementary material for: Echocardiographic Indices in Patients with End-Stage Renal Disease and Their Association with Hemodialysis-to-Hemodiafiltration Transfer: A Prospective Observational Study
Source: Medicina (Kaunas). 2024 Sep 20;60(9):1537. doi: 10.3390/medicina60091537 (PMC11434155; doi:10.3390/medicina60091537)
Supplement: Supplementary file 1 [file medicina-60-01537-s001.zip › medicina-3095932-supplementary.docx]

**Supplementary Files:**

**Supplementary Figure 1.** Flow diagram.


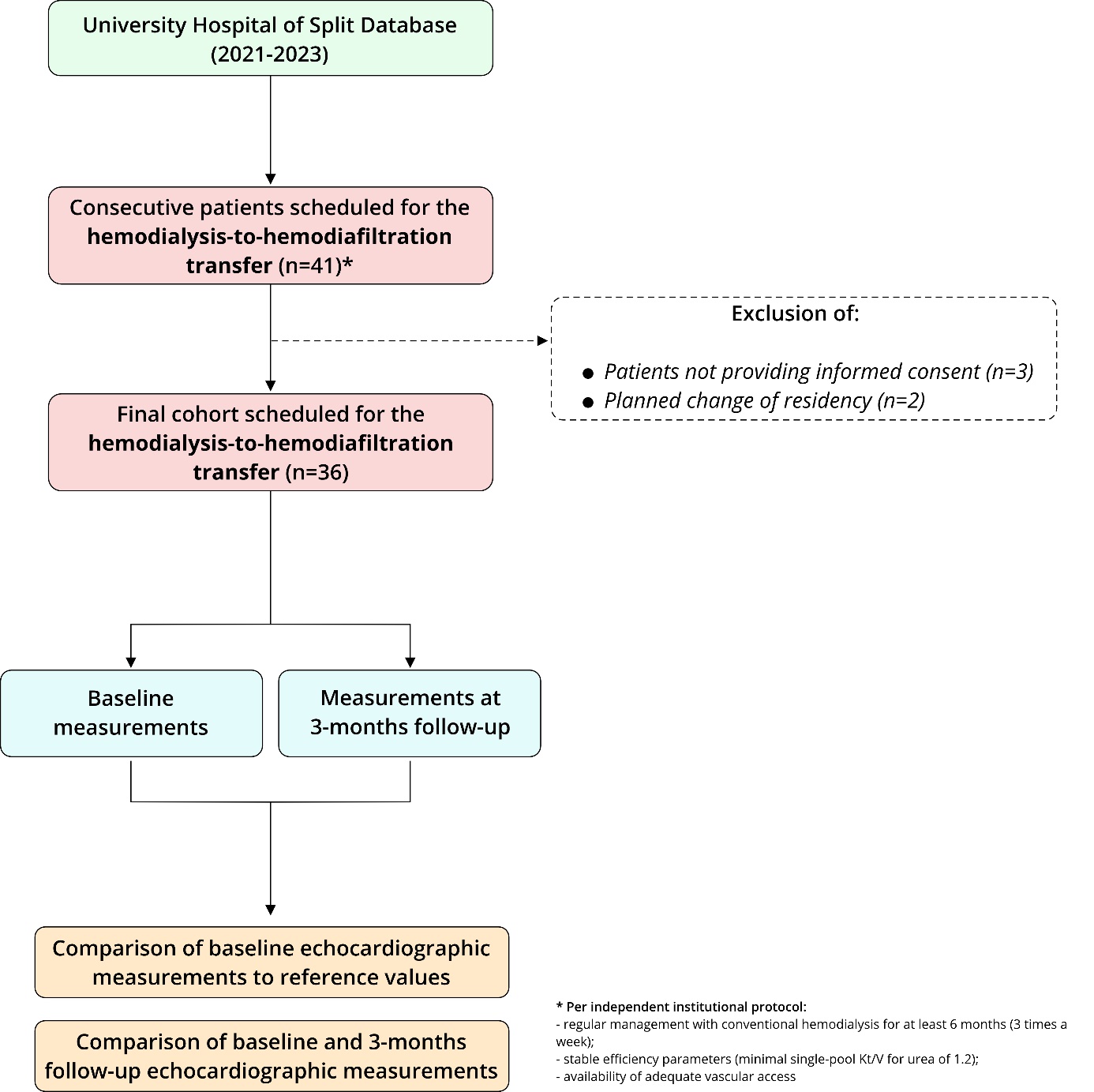


**Abbreviations:** None.

**Supplementary Figure 2.** Comparison of baseline contemporary echocardiographic measures of left ventricular myocardial work and longitudinal strain to reference values from the general population (mean and 95% confidence intervals).

**
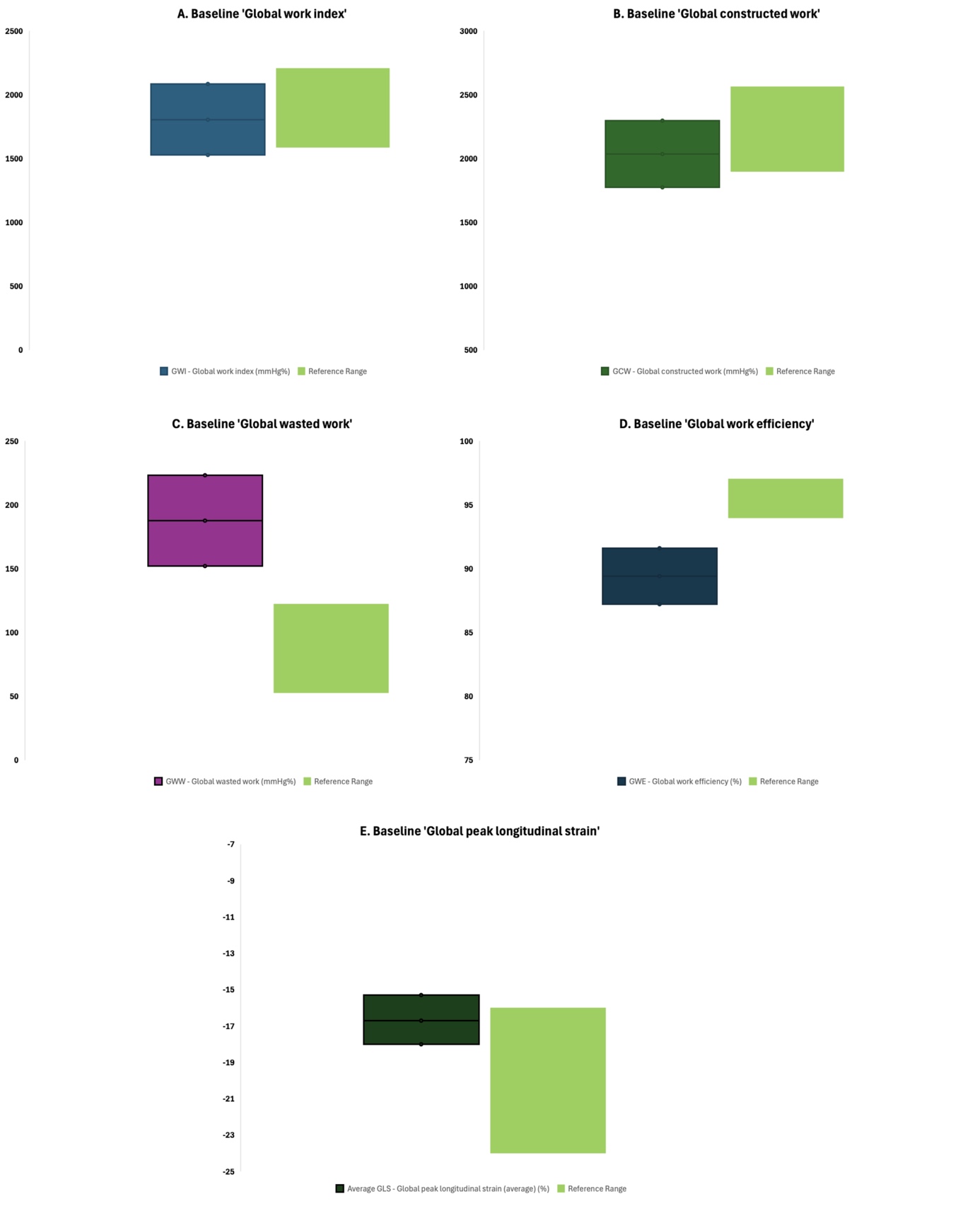
**

Data are presented as mean and 95% confidence intervals.

**Abbreviations:** none.

**Supplementary Table 1.** Baseline characteristics of the study sample.

| **Variables** | **Study sample (N=36)** |
| --- | --- |
| **Age (years)** | 62.0 (56.0-71.0) |
| **Female sex** | 41.7% |
| **BMI (kg/m^2^)** | 25.4 (22.5-28.4) |
| **BSA (m^2^)** | 1.9 (1.8-2.0) |
| **Diuresis (ml per day)** | 500.0 (0-1000.0) |
| **Dialysis duration (years)** | 4.0 (2.0-5.0) |
| **Dialysis access** |  |
| AVF | 66.7% |
| Tunneled CVC | 33.3% |
| **Comorbidities** |  |
| **Arterial hypertension** | 86.1% |
| **Diabetes mellitus** | 25.0% |
| **Prior CVI** | 16.7% |
| **Prior AMI** | 25.0% |
| **CABG** | 16.7% |
| **PAD** | 30.5% |
| **Atrial fibrillation** | 5.6% |
| **Hypothyroidism** | 13.9% |
| **Liver cirrhosis** | 0.0% |
| **COPD/asthma** | 5.6% |
| **Previous parathyroidectomy** | 8.3% |
| **History of malignant disease** | 8.3% |
| **Monoclonal gammopathy** | 5.6% |
| **IBD** | 5.6% |
| **Polycystic kidney disease** | 11.0% |

Data are expressed as number (percent) or median (interquartile range).

**Abbreviations:** AMI – acute myocardial infarction; AVF – arteriovenous fistula; BMI – body mass index; BSA – body surface area; CABG – coronary artery bypass grafting; COPD – chronic obstructive pulmonary disease; CVC – central venous catheter; CVI – cerebrovascular incident; IBD – inflammatory bowel disease; PAD – peripheral arterial disease.

**Supplementary Table 2.** Comparison of **baseline contemporary** echocardiographic measures of **left ventricular myocardial work and longitudinal strain** to reference values from the general population

| **Echocardiographic parameters** | **Study sample**  **(baseline – hemodialysis treatment)** | **Reference values** |
| --- | --- | --- |
| **GWI - Global work index (mmHg%)** | 2001.0 (1368.0-2148.0) | 1588.0-2204.0 |
| **GCW - Global constructed work (mmHg%)** | 2084.5 (1628.0-2440.0) | 1901.0-2563.0 |
| **GWW - Global wasted work (mmHg%)** | 179.0 (148.0-217.0) * | 53.0-122.2 |
| **GWE - Global work efficiency (%)** | 90.0 (89.0-92.0) * | 94.0-97.0 |
| **Average GLS - Global peak longitudinal strain (average) (%)** | -16.0 (-19.0-[-14.0]) * | -24.0-[-16.0] |

Data are expressed as median (interquartile range).

* Outside of the reference range.

**Abbreviations:** none.

**Supplementary Table 3.** Baseline laboratory parameters of the study sample

| **Laboratory parameters** | **Baseline**  **(hemodialysis treatment)** | **3-month follow up**  **(hemodiafiltration treatment)** | **p-value** |
| --- | --- | --- | --- |
| **WBC (x10^9^/L)** | 6.1 (5.1-6.9) | 5.9 (4.8-6.4) | 0.267 |
| **RBC (x10^12^/L)** | 3.9 (3.7-4.0) | 3.4 (3.2-3.9) | 0.012 |
| **Hgb (g/L)** | 119.0 (114.0-122.0) | 109.0 (98.0-118.0) | 0.009 |
| **MCV (fL)** | 94.2 (89.5-97.7) | 93.1 (89.5-97.8) | 0.991 |
| **Platelets (x10^3^/L)** | 180.0 (130.0-254.0) | 182.0 (126.0-280.0) | 0.982 |
| **CRP (mg/L)** | 3.0 (1.6-5.5) | 3.3 (1.4-5.5) | 0.668 |
| **Uric acid (μmol/L)** | 347.0 (321.0-378.0) | 323.0 (270.0-352.0) | 0.153 |
| **Proteins (g/L)** | 66 (63.0-71.0) | 65.0 (62.0-69.0) | 0.343 |
| **Albumin (g/L)** | 41.8 (40.6-43.0) | 41.8 (40.6-43.0) | 0.203 |
| **PTH (pmol/L)** | 24.2 (17.6-37.7) | 31.9 (16.5-40.6) | 0.590 |
| **NT-pro-BNP (pg/mL)** | 6275.0 (3703.0-13787.0) | 5634.0 (2339.0-12774.8) | 0.524 |
| **Ferrum (μmol/L)** | 12.0 (9.0-15-0) | 12.0 (10.0-16.0) | 0.903 |
| **Feritin (ng/mL)** | 462.0 (286.0-580.0) | 351.0 (255.0-504.0) | 0.262 |

Data are expressed as median (interquartile range) and compared using the Mann Whitney U test.

**Abbreviations:** BUN – blood urea nitrogen; CRP – C-reactive protein; Hgb – hemoglobin; MCV – mean corpuscular volume; NT-pro-BNP – N terminal pro-brain natriuretic peptide; PTH – parathyroid hormone; RBC – red blood cell; WBC – white blood cell

**Supplementary Table 4.** Relevant laboratory measurements regarding the dialysis treatment: pre-dialysis and post-dialysis (at study onset and 3-month follow-up)

| **Laboratory parameters** | | **Baseline**  **(hemodialysis treatment)** | **3-month follow up**  **(hemodiafiltration treatment)** | **p-value** |
| --- | --- | --- | --- | --- |
| **β2 microglobulin (mg/L)** | **Pre-dialysis** | 40.3 (30.3-42.6) | 25.0 (23.2-27.5) | <0.001 |
|  | **Post-dialysis** | 46.8 (31.4-53.8) | 9.6 (7.3-10.7) | <0.001 |
| **Troponin (ng/L)** | **Pre-dialysis** | 75.0 (50.8-105.9) | 67.4 (58.3-88.0) | 0.893 |
|  | **Post-dialysis** | 75.3 (58.7-137.6) | 76.3 (63.9-94.7) | 0.813 |
| **Kt/V** |  | 1.2 (1.2-1.3) | 1.4 (1.2-1.5) | 0.024 |

Data are expressed as median (interquartile range) and compared using the Mann Whitney U test.

**Abbreviations:** Kt/V – mathematical relationship between the rate of urea removal (K) times treatment duration (t) divided by the volume of distribution for urea (V).
